# Quality of life following a false positive mammogram.

**DOI:** 10.1038/bjc.1990.430

**Published:** 1990-12

**Authors:** I. T. Gram, E. Lund, S. E. Slenker

**Affiliations:** Institute of Community Medicine, University of Tromsö, Norway.

## Abstract

To assess how women regard having had a false positive mammogram screening exam, and the influence that this had on their quality of life, 126 such women were interviewed. Their responses were compared to those of 152 women randomly selected among screenees with a negative exam. Eighteen months after the screening the reported prevalence of anxiety about breast cancer was 29% among women with a false positive and 13% among women with a negative screening mammogram (P = 0.001). Of 30 women biopsied, 8 (27%) had pain in the breast and 10 (33%) had reduced sexual sensitivity. A false positive mammogram was described by 7 (5%) of the women as the worst thing they ever had experienced. However, most women with a false positive result regarded this experience, in retrospect, as but one of many minor stressful experiences creating a temporary decrease in quality of life. They report the same quality of life today as women with negative screening results and 98% would attend another screening. Even so, false positive results are a matter of concern, and efforts should be made to minimise this cost whenever a screening programme is conducted.


*Br. J. Cancer* (1990), **62**, 1018–1022 © Macmillan Press Ltd., 1990

### Quality of life following a false positive mammogram

I.T. Gram[1], E. Lund[1] & S.E. Slenker[2]

[1]*Institute of Community Medicine, Box 417, University of Tromsö, N-9001 Norway; and* [2]*Department of Health Behavior, School of Public Health, University of Alabama at Birmingham, AL 35294, USA.*

**Summary** To assess how women regard having had a false positive mammogram screening exam, and the influence that this had on their quality of life, 126 such women were interviewed. Their responses were compared to those of 152 women randomly selected among screenees with a negative exam. Eighteen months after the screening the reported prevalence of anxiety about breast cancer was 29% among women with a false positive and 13% among women with a negative screening mammogram ($P = 0.001$). Of 30 women biopsied, 8 (27%) had pain in the breast and 10 (33%) had reduced sexual sensitivity. A false positive mammogram was described by 7 (5%) of the women as the worst thing they ever had experienced. However, most women with a false positive result regarded this experience, in retrospect, as but one of many minor stressful experiences creating a temporary decrease in quality of life. They report the same quality of life today as women with negative screening results and 98% would attend another screening. Even so, false positive results are a matter of concern, and efforts should be made to minimise this cost whenever a screening programme is conducted.

The reduced breast cancer mortality found in several major studies (Shapiro *et al.*, 1982; Collette *et al.*, 1984; Verbeek *et al.*, 1984; Tabar *et al.*, 1985, 1989; Palli *et al.*, 1986) is the rationale for screening with mammography. In order to justify the continued use of a screening procedure, subjects correctly classified as positive at screening should receive a benefit. However, the magnitude of the reduction in breast cancer resulting from screening has been questioned, and issues regarding adverse effects of breast screening have been raised (Skrabanak, 1985, 1988; Wright, 1986; Eddy, 1988; Devitt, 1989).

So far, breast screening has not been found to increase psychiatric morbidity as measured by the General Health Questionnaire, neither among women with negative (Dean *et al.*, 1986) nor false positive screening results (Ellman *et al.*, 1989). In the Canadian National Breast Screening Study (Baines *et al.*, 1990) 93% of the women, receiving either annual mammography or physical examinations for three or four years, reported this as a positive experience. Women's attitudes and expectations based upon their own experiences are important aspects of the screening issue that need to be addressed further. This study set out to investigate how women regard having had a false positive result at a mammography screening, and whether the experience has consequences for their attitude toward mammography and long-term quality of life.

### Materials and methods

### *Screening/work-up examination*

The mammography screening was a part of a health survey carried out in Tromsö, Norway 1986/87. Women aged 40 or older ($n = 4{,}323$), were offered a free mammogram, and 85% of these women had their mammogram taken. The women were told that only those with an abnormal mammogram would be notified by mail within three weeks. Altogether 193 (5%) of the screenees were selected for a work-up mammographic examination, and of these 61 were subsequently referred to a surgeon. Altogether 40 (1%) women underwent biopsy, mostly as hospital inpatients, and ten new cases of breast cancer were diagnosed. Details of the screening and case finding procedures are given elsewhere (Gram *et al.*, 1989). Fourteen women were ineligible for the present study (two lost to migration before work-up, ten with a new and two with a previous diagnosis of breast cancer). The remaining 179 women with a false positive screening result formed the study group.

### *Questionnaire*

A questionnaire concerning attitudes toward mammography, anxiety about having breast cancer and a request for a future interview were mailed to the study group six months after the screening mammogram. The questionnaire was also mailed to the following three groups: a random sample of 250 women selected from women with a negative screening result (reference sample), a random sample of 250 women not invited to screening living in the nearby city of Harstad (population sample) and women invited who did not attend (non-attenders, $n = 670$) (Figure 1). In the study group 89% completed the questionnaire. The corresponding completion rates for the eligible women in the reference group was 84%, among non-attenders 43%, and in the population sample 66%. Women completing the questionnaire although migrated ($n = 31$, non-attenders) are included in the analysis. The women in the combined comparison groups were within the same age range.

### *Interview*

Women in the study and reference group who had indicated that they would allow an interview were contacted about 1 year after returning their questionnaire. Women who did not show up were mailed a new time for appointment. Those still not responding were approached by telephone and their

**Figure 1** Flow chart of the mammography screening in Tromsö, Norway 1986/87 and questionnaire response status among the four comparison groups.

Correspondence: I.T. Gram, Department of Epidemiology, Tidwell Hall, Room 201, University of Alabama at Birmingham, AL 35294, USA.
Received 24 April 1990; and in revised form 3 July 1990.

reason for lack of response sought. All women were interviewed in person by one of four female interviewers.

The interview comprised open-ended, dichotomous, scaled and paired comparison questions. Two cards showing different alternatives were handed the respondent when comparisons were used. Members of the study group were asked to recall the time interval between being informed of their abnormal mammogram result and the subsequent notification of their results from the work-up. For brevity this period is referred to in the text as the work-up period. Members of the reference group were asked to recall the 3 weeks subsequent to the screening, when they did not know the result of their screening mammogram. For brevity this period is referred to in the text as the screening period. As an indicator of well-being a ladder scale with ten rungs, derived from the Self Anchoring Scale of Hadley Cantril (Cantril, 1965) was used. The top rung was labelled 'Best life I could expect to have' and the bottom rung 'Worst life I could expect to have'. The respondents were asked to rate themselves today. Afterwards, the study group rated themselves in the work-up period and the reference group in the screening period. The study group was questioned as to whether they would be willing to go through a similar work-up if it were free, or to pay any amount of money to get a reviewed and final result of the screening mammogram the next day without further assessments, assuming this was technically feasible. The reference group cited what they would pay to get the result of the screening mammogram the following day. As an indicator of willingness to trade longevity for quality of life, some questions derived from the proportional trade-off method were used (Weinstein *et al.*, 1980). Members of the study group were asked if they would trade-off, in the following order, 21, 1, 7, or 14 of their last days of life (assuming a life-span of 79 years and remaining healthy) to avoid going through the work-up period. Women in the reference group were asked the same question regarding the screening period.

Spontaneous comments on the different questions were recorded. The women were encouraged to talk freely at the end of the interview which took about 30 min to complete. The analyses were performed using the Pearson $\chi^2$ statistic and $t$ test procedures available in the SAS statistical package (SAS Version 6). Results were considered statistically significant with a $P$ value of 0.05 or less.

### Results

Analysis of questionnaire responses 6 months after the screening revealed a prevalence of anxiety about breast cancer in the study group of 40% and in the reference group of 22% ($P<0.001$) (Table I). The corresponding prevalence was 21% in the non-attenders group and 33% in the population group. The latter was significantly higher compared with the reference group ($P=0.03$). Eighteen months after the screening the prevalence of anxiety about breast cancer was 29% in the study group and 13% in the reference group ($P=0.001$).

Among the women completing the questionnaire 90% in the study group and 88% in the reference group indicated their willingness to be interviewed (Table II). When invited, 88% of the former and 83% of the latter group attended. Table III shows that the two groups were similar with respect to a number of selected characteristics at the time of the mammography screening.

Neither of the groups interviewed had changed their frequency of visits to health professionals during the preceding year, compared to what they reported at the time of the screening. No significant differences were found between the study and reference groups with respect to their being easily worried, suffering from sleeplessness, taking sleeping pills or sedatives, or frequency of breast self-examination (results not shown in tables).

Table IV shows that both groups had an average state of well-being of 7.7 on the Ladder scale at the time of the interview. The study group recalled a significant decrease in

**Table I** Prevalence of anxiety about breast cancer reported by group according to questionnaire and interview

| | *Group* | *Prevalence (%) (95% CI)* | $\chi^2$ |
|---|---|---|---|
| Questionnaire 6 months after screening | Study ($n=151$) | 40 (32–48) | 12.6*** |
| | Reference ($n=206$) | 22 (17–28) | |
| | Non-attenders ($n=230$) | 21 (16–27) | 0.1 n.s. |
| | Population ($n=155$) | 33 (26–40) | 5.0* |
| Interview 18 months after screening | Study ($n=126$) | 29 (21–37) | 10.2** |
| | Reference ($n=152$) | 13 (7–18) | |

n.s. Not significantly different from reference group. *Significantly different from reference group ($P=0.03$). **Significantly different from reference group ($P=0.001$). ***Significantly different from reference group ($P<0.001$).

**Table II** Interview response status (%) of women completing the questionnaire by group

| | *Group* | |
|---|---|---|
| *Response status* | *Study (n = 160) %* | *Reference (n = 209) %* |
| Declined | 16 (10) | 26 (12) |
| Not attended | 18 (11) | 31 (15) |
| Attended | 126 (79) | 152 (73) |

**Table III** Selected attributes for women in study and reference group at the time of the screening given as mean (s.e.) or per cent (%)

| | *Group[a]* | |
|---|---|---|
| | *Study n = 126* | *Reference n = 152* |
| Age (years) | 46.4 (0.4) | 47.2 (0.4) |
| Years of education | 9.9 (0.3) | 10.0 (0.3) |
| Number of children | 2.3 (0.1) | 2.6 (0.1) |
| Married (%) | 83 | 85 |
| Full time work (%) | 54 | 48 |
| Children under 10 years (%) | 12 | 16 |
| Health condition well (%) | 81 | 73 |
| Headache monthly or more (%) | 51 | 50 |
| Able to cope with problems last two weeks if any (%) | 82 | 80 |
| Visits last year to | | |
| general practitioner | 1.6 (0.2) | 1.6 (0.1) |
| outpatient department | 0.6 (0.1) | 0.5 (0.1) |
| physiotherapist | 1.8 (0.5) | 2.1 (0.5) |

[a]Some values are based on fewer than the total number due to missing values.

**Table IV** Average state of well-being reported on the Ladder Scale at time of interview and during work-up[a] and screening[b] period by group

| | *Group* | |
|---|---|---|
| *Time period* | *Study (n = 126)* | *Reference (n = 152)* |
| Interview | 7.7 | 7.7 |
| Result unknown | 5.5[a]* | 7.2[b] n.s. |

[a]Interval between being informed of their abnormal mammogram result and subsequent notification of their result from the work-up. [b]Three weeks subsequent to the screening, when they did not know the result of their screening mammogram. *Significantly different from time of interview ($P<0.001$). n.s., Not significantly different from time of interview.

**Table V** Women (%) in study and reference group considering listed minor events to be more stressful to them than respectively the work-up[a] and screening[b] period

| | Group | | |
|---|---|---|---|
| | Study | | |
| | Biopsy | | Reference |
| | yes | no | |
| | (n = 29) | (n = 94) | (n = 152) |
| Minor events | % | % | % |
| Headache one day | 24* | 60* | 83 |
| Gastric flu one day | 38* | 69* | 95 |
| Rain three weeks of vacation | 38* | 74* | 97 |
| Sprain the ankle | 41* | 72* | 98 |

[a]Interval between being informed of their abnormal mammogram result and subsequent notification of their result from the work-up. [b]Three weeks subsequent to the screening, when they did not know the result of their screening mammogram. *Significantly different from reference group ($P<0.001$).

**Table VI** Highest amount of money ($) the women would pay to attend another mammography screening given as mean (s.e.) and median (range), by group

| | Group | | |
|---|---|---|---|
| | Study | | |
| | Biopsy | | Reference |
| | yes | no | |
| Amount of money in US | (n = 30) | (n = 94) | (n = 147) |
| dollars | $ | $ | $ |
| Mean (s.e.) | 60 (12) | 70* (9) | 46 (4) |
| Median (range) | 32 (0–286) | 43 (0–429) | 29 (0–143) |

*Significantly different from reference group ($P = 0.02$).

**Table VII** Highest amount of money ($) the women would pay to get the results of the work-up[a] and screening[b] the next day, given as mean (s.e.) and median by group

| | Group | | |
|---|---|---|---|
| | Study | | |
| | Biopsy | | Reference |
| | yes | no | |
| Amount of money in US | (n = 30) | (n = 91) | (n = 152) |
| dollars | $ | $ | $ |
| Mean (s.e.) | 66 (12)* | 32 (6)* | 10 (2) |
| Median (range) | 29 (0–286) | 14 (0–429) | 0 (0–143) |

[a]Get a reviewed and final result of the screening mammogram the next day without further assessments. [b]Get the result of the screening mammogram the next day. *Significantly different from reference group ($P<0.001$).

**Table VIII** Women (%) reporting how many days of their lives[a] they would trade off in exchange for not experiencing the work up[b] or screening[c] period another time, by group

| | Group | | |
|---|---|---|---|
| | Study | | |
| | Biopsy | | Reference |
| | yes | no | |
| | (n = 29) | (n = 93) | (n = 148) |
| No. of days | % | % | % |
| None | 24* | 35* | 69 |
| 1, 7, 14 | 10 | 11 | 7 |
| 21 | 66* | 54* | 24 |

[a]Assuming a life-span of 79 years and remaining healthy. [b]Interval between being informed of their abnormal mammogram result and subsequent notification of their result from the work-up. [c]Three weeks subsequent to the screening, when they did not know the result of their screening mammogram. *Significantly different from reference group ($P<0.0001$).

their state of well-being during the work-up period ($P = 0.0001$). A slight decrease in well-being reported by the reference group was not statistically significant.

In the study group 95 (80%) of 118 indicated the duration of the work-up period to be 4 weeks or less. The women's perceptions of the length of the work-up period was longer than that documented in the hospital files (Wilcoxon signed rank test, $P = 0.05$). Eighty (63%) of the women reported that they had been anxious during the work-up period. Among them 14 (11%) claimed they had less capacity for work until learning the result of the work-up, while 19 (15%) reported they had this problem only on some days. In the reference group 24 (16%) said they were anxious about the result of their screening mammogram, and one of them reported having less capacity for work because of this anxiety.

Thirty-one per cent in the study group and 38% in the reference group considered themselves to be frequently subjected to stress ($P = 0.3$). Events occurring within the family such as death, serious disease, conflicts and major accidents were incidents perceived by the study group to involve more strain than the work-up period. Having a pelvic examination, visiting a dentist and waiting for medical test results were situations most frequently described as subjecting them to a degree of stress similar to that of the work-up period. Six (5%) of 117 women said they had never suffered anything worse than having a false alarm at the mammography screening. Five of these women had undergone biopsy. However, all six said they would attend another screening with mammography.

Table V shows that about 40% of biopsied women regarded minor stressful events such as suffering from gastric flu or spraining an ankle as probably causing them more inconvenience and stress than the work-up period did. Among women not having a diagnostic biopsy about 70% considered the mentioned events as probably more traumatising than the work-up period was. Most of the women, but not all, in the reference group considered the screening period as less stressful than the events they compared it to.

Women in the study group not biopsied were on the average, willing to pay $70 to attend another screening (Table VI). This was $10 more than the women biopsied were willing to pay ($P = 0.5$) and $24 more than women in the reference group were willing to pay ($P = 0.02$). While answering this question, many women made their own comparison saying they would pay a cost equal to that of a visit to a physician ($7), to a dentist ($70) or of a car repair ($150). Table VII shows that biopsied women would be willing to pay the highest amount of money ($66) to get the result of the examination the next day without any further assessments. Only one of the women biopsied was willing to pay more than $150 to avoid this experience again. In the reference group 100 (66%) claimed that they would rather wait for 3 weeks than pay anything to get the result the next day.

However, as shown in Table VIII, 76% of biopsied women reported to be willing to trade off days of their lives in the future, assuming this could spare them another work-up period. Among the women in the study group not subjected to surgery 65% were willing to trade off days of life to avoid the work-up period. In the reference group 31% said they would trade off days of life in exchange for having the result of the screening mammogram the next day.

Of the 30 women who underwent biopsy, eight (27%) had pain from the scar, while ten (33%) had reduced sexual sensitivity in the breast. Three (2%) women described that having a false alarm at the screening subsequently had an overall bad influence on their lives. For two of them this was due to trouble from the scar caused by surgery. The third woman said she had become more anxious about breast cancer. In the study group 44% claimed that the experience of going through the screening and the work-up had an overall positive impact on their lives. However, these women said more often than the rest of the study group that they had been anxious in the work-up period ($P = 0.04$). In the reference group 53% claimed that the mammography screening had an overall positive impact on their lives. The remaining women in both groups considered these experiences of minor significance and reported no overall impact. Only three (1%) of 278 women did not want to participate if they were again offered a free screening with mammography, while another 11 (4%) said they would not attend if they had to pay.

### Discussion

This study shows that most women with a false positive result at a mammography screening regard this experience, in retrospect, as but one of many minor stressful experiences in their lives. It also demonstrates that these women are in favour of attending another screening, and that they report the same quality of life today as women with negative screening results.

One long-term adverse effect found in this study is the physical morbidity, i.e. pain and reduced sexual sensitivity described by some of the women subjected to surgery. This negative impact on sexuality was also commented on by some of the women participating in the Canadian study (Baines *et al.*, 1990).

Another effect found in our study is that women with a false positive screening result have a higher prevalence of anxiety about breast cancer compared with women with a negative screening mammogram. The high prevalence of anxiety about breast cancer reported by the population group not exposed to mammography screening indicates that this anxiety is widespread in the general population. The results from the questionnaire suggest that the screening is generating an increase in this prevalence among women in the false positive group and a decrease among women in the negative result group. Time seems to have an impact on level of anxiety about breast cancer, since both groups have a decreased prevalence at 18 months compared with 6 months after the screening. Of the women attending Edinburgh Breast Screening Clinic (Dean *et al.*, 1986) 40% said they sometimes worried about the possibility of having breast cancer before the screening. This proportion did not change 6 months after the screening. Among women attending the screening program in Canada (Baines *et al.*, 1990) for 3 or 4 years, only 5% reported being anxious and another 5% that this varied. Sixty-one per cent of the women offering explanations for their anxiety said it was because they had been referred to the review clinic. In spite of this, the responses to the question about anxiety induced by screening, were not found to differ significantly by review status.

In our study it is noteworthy that women willing to pay the highest amount of money to attend another screening are found among those who experienced a positive screening test, but who did not go through diagnostic surgery. It is also notable that a substantial proportion of the study group reported that this experience had a positive impact on their lives. Some of them stated explicitly that they were grateful for this experience, because they found life more precious afterwards. However, it seems unreasonable to put this on the positive side of the balance sheet of a screening, since first the fear, then the relief, are induced by the same screening. Nevertheless, the data suggest that women correctly classified as negative have gained a benefit from the screening, as the majority report that the screening had an overall positive impact on their lives.

With regard to the question of trading longevity, an inconsistency appeared. That is, some biopsied women would rather go through another operation than trade a single day in the future, while others were willing to trade 3 weeks of their lives in exchange for having the screening result the next day. In our survey, answers to these questions do not seem to reflect what they were intended to measure, that is how much stress the women had been through. It rather reflects main differences in attitude toward longevity. The following two viewpoints emerged from spontaneous comments during the interview. When an age of 79 years was assumed, it mattered little to the women if they were alive 21 days more or less. The other one was that if healthy, even 1 day that far away was too much to trade to avoid a reduction in quality of life today.

The fact that women recalled the duration of the work-up period to be longer than it probably was, can be interpreted as an indirect measure of the unpleasantness of the work-up period. This difference, however, may also be explained by missing information on later visits in the hospital files.

Since the purpose of this investigation was to focus on the consequences that a false positive result has on women attending a screening, subjects with a negative mammogram result were chosen as a reference group. This is not fully satisfactory since the two groups have to compare different experiences when answering some of the questions. The interview method was selected to allow observation of how the women responded to the questions. Based on hypothetical situations, some answers depend on the women's ability to abstract comparisons. A potential weakness of the method applied is the possible risk of bias due to the attitudes of the interviewers. An interesting observation is that all women subjected to surgery agreed to and were available for interview, as opposed women not subjected to surgery. This fact creates a selection bias toward emphasising the opinions of biopsied women more than their true proportion among women with false positive mammogram should imply.

The increased morbidity induced by mammography screening has led some authors advocate the abandonment (Wright, 1986) or discouragement (Devitt, 1989) of such screening before the age of 60. This paper is an attempt to evaluate the magnitude of this morbidity. Even if the women with a false alarm at the screening report the same quality of life today as do women with negative screening mammogram, our data suggest that some of them will suffer from undesirable long-term effects, and a small proportion will experience this as subsequently having an overall bad influence on their lives. Efforts should be made to minimise this cost whenever a screening programme is conducted.

Dr Gram is a research fellow of the Norwegian Cancer Society. We thank all women concerned for their co-operation in first completing the questionnaires and then attending the interview. We thank Dr P. Cole for valuable comments during the preparation of the manuscript. Financial support was given by the Norwegian Cancer Society and the Aakre Foundation.

### References

BAINES, C.J., TO, T. & WALL, C. (1990). Women's attitudes after participating in the National Breast Screening Study. *Cancer*, **65**, 1663.

CANTRIL, H. (1965). *The Pattern of Human Concerns*. Rutgers University Press: New Brunswick.

COLLETTE, H.J.A., DAY, N.E., ROMBACH, J.J. & DE WAARD, F. (1984). Evaluation of screening for breast cancer in a non-randomised study (the DOM-project) by means of a case-control study. *Lancet*, **i**, 1224.

DEAN, C., ROBERTS, M., FRENCH, K. & ROBINSON, S. (1986). Psychiatric morbidity after screening for breast cancer. *J. Epidemiol. Comm. Health*, **40**, 71.

DEVITT, J.E. (1989). False alarms of breast cancer. *Lancet*, **ii**, 1257.

EDDY, D.M., HASSELBLAD, V., MCGIVNEY, W. & HENDEE, W. (1988). The value of mammography screening in women under age 50 years. *JAMA*, **259**, 1512.

ELLMAN, R., ANGELI, N., CHRISTIANS, A., MOSS, S., CHAMBERLAIN, J. & MAGUIRE, P. (1989). Psychiatric morbidity associated with screening for breast cancer. *Br. J. Cancer*, **60**, 781.

GRAM, I.T., LUND LARSEN, P.G., STÖRMER, J. & ROSENLUND, A.F. (1989). Mammografiscreening i Tromsø. *Tidsskr. Nor. Laegeforen*, **109**, 1040. (Abstract in English.)

PALLI, D., DEL TURCO, M.R.D., BIUATTI, E. & 4 others (1986). A case-control study of the efficacy of a non-randomised breast cancer screening programme in Florence (Italy). *Int. J. Cancer*, **38**, 501.

TABAR, L., FAGERBERG, G., DUFFY, S.W. & DAY, N.E. (1989). The Swedish two county trial of mammographic screening for breast cancer: recent results and calculation of benefit. *J. Epidemiol. Comm. Health*, **43**, 107.

TABAR, L., FAGERBERG, G., GAD, A. & 9 others (1985). Reduction in mortality from breast cancer after mass screening with mammography. *Lancet*, **i**, 829.

SAS INSTITUTE (1987). SAS/STAT Guide for Personal Computers, Version 6 Edition. SAS Institute Inc.: Gary, NC.

SHAPIRO, S., VENET, W., STRAX, P.H., VENET, L. & ROESER, R. (1982). Ten-to-fourteen year effect of screening on breast cancer mortality. *J. Natl Cancer Inst.*, **69**, 349.

SKRABANEK, P. (1985). False premises and false promises of breast cancer screening. *Lancet*, **ii**, 316.

SKRABANEK, P. (1988). The debate over mass mammography in Britain. The case against. *Br. Med. J.*, **297**, 971.

VERBEEK, A.L.M., HENDRIKS, J.H.C.L., HOLLAND, R., MRAVUNAC, M., STURMANS, F. & DAY, N.E. (1984). Reduction of breast cancer mortality through mass screening with modern mammography: first results of the Nijmegen Project, 1975–81. *Lancet*, **i**, 1222.

WEINSTEIN, M.C., FINEBERG, H.V., ELSTEIN, A.S. & 4 others (1980). *Clinical Decision Analysis*, Chapter 7, p. 215. Saunders: New York.

WRIGHT, C. (1986). Breast cancer screening; a different look at the evidence. *Surgery*, **100**, 594.


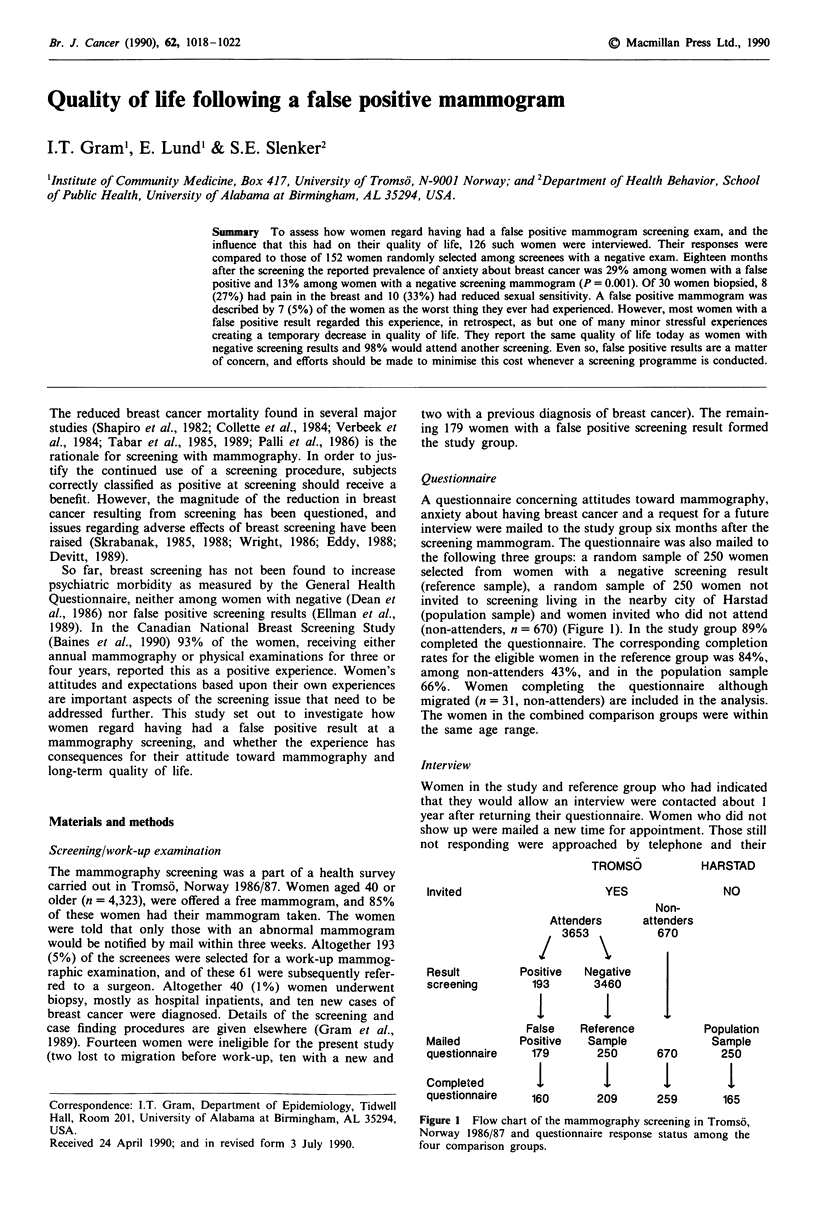

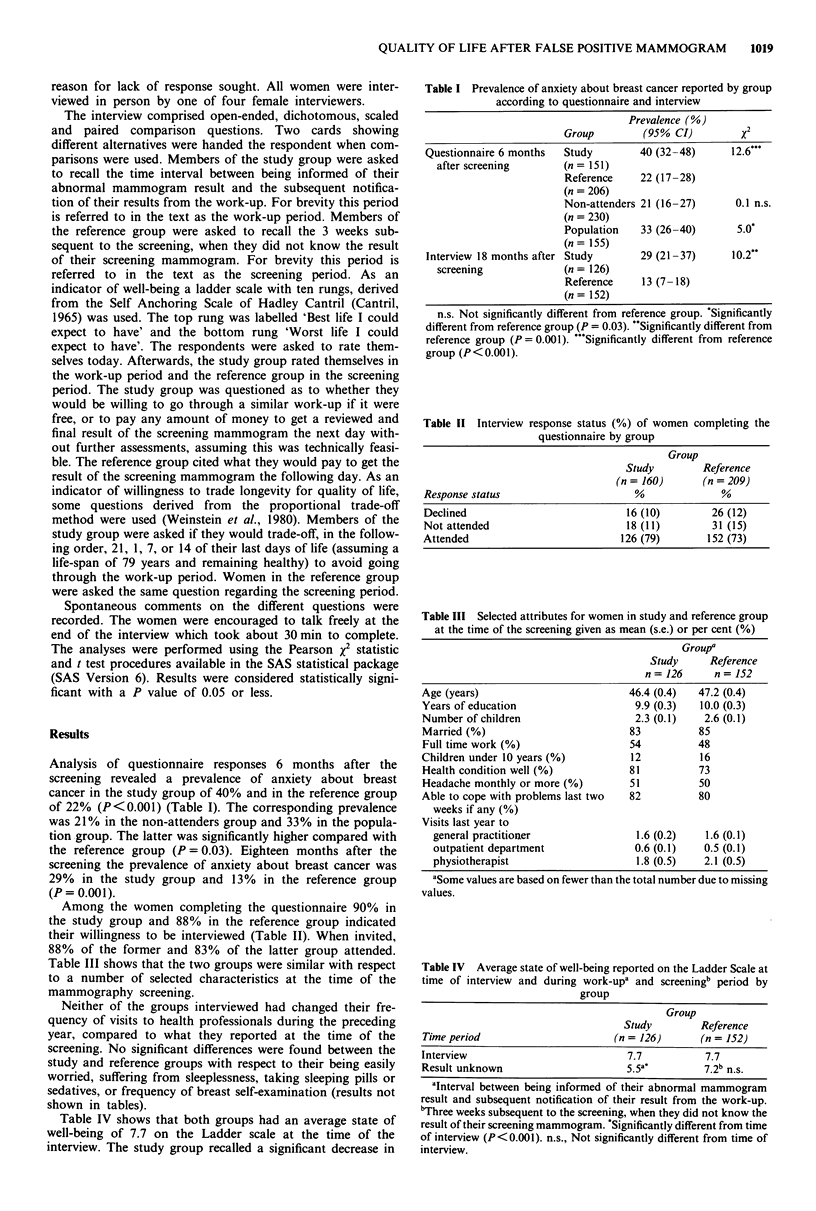

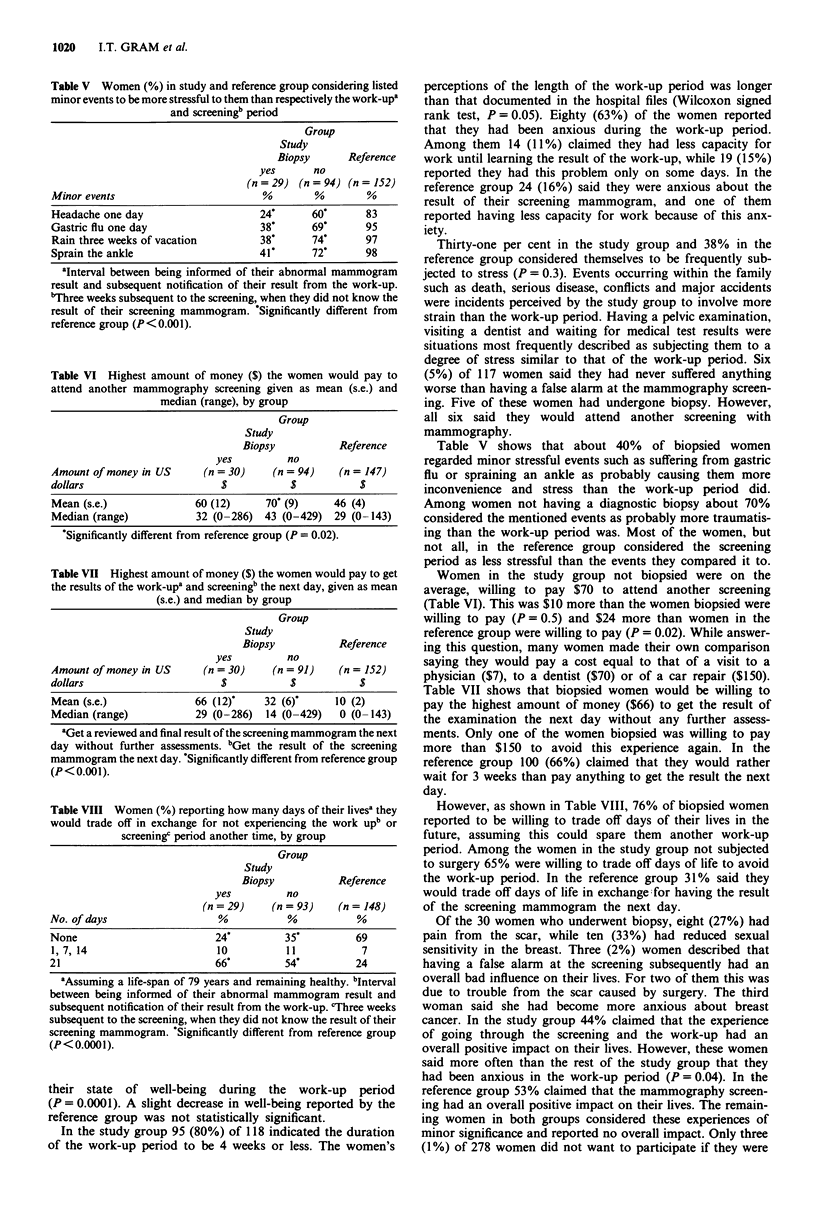

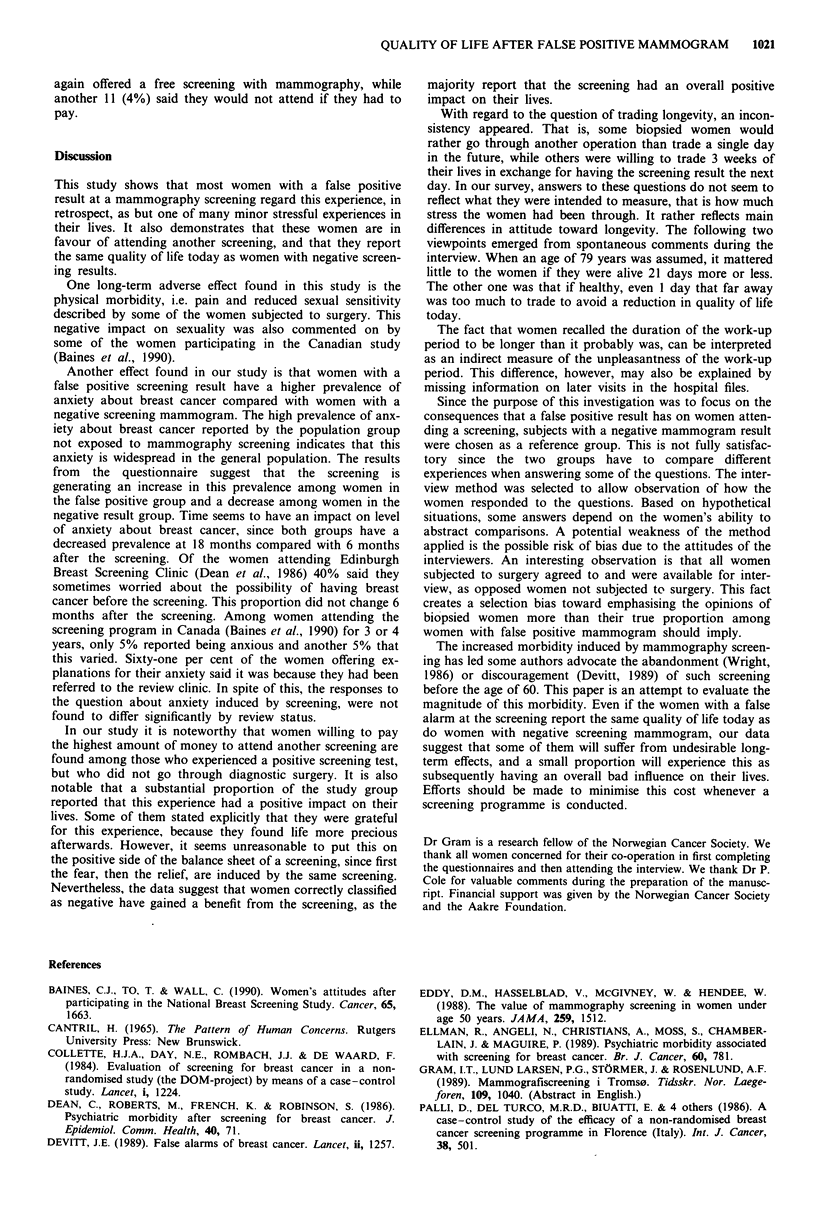

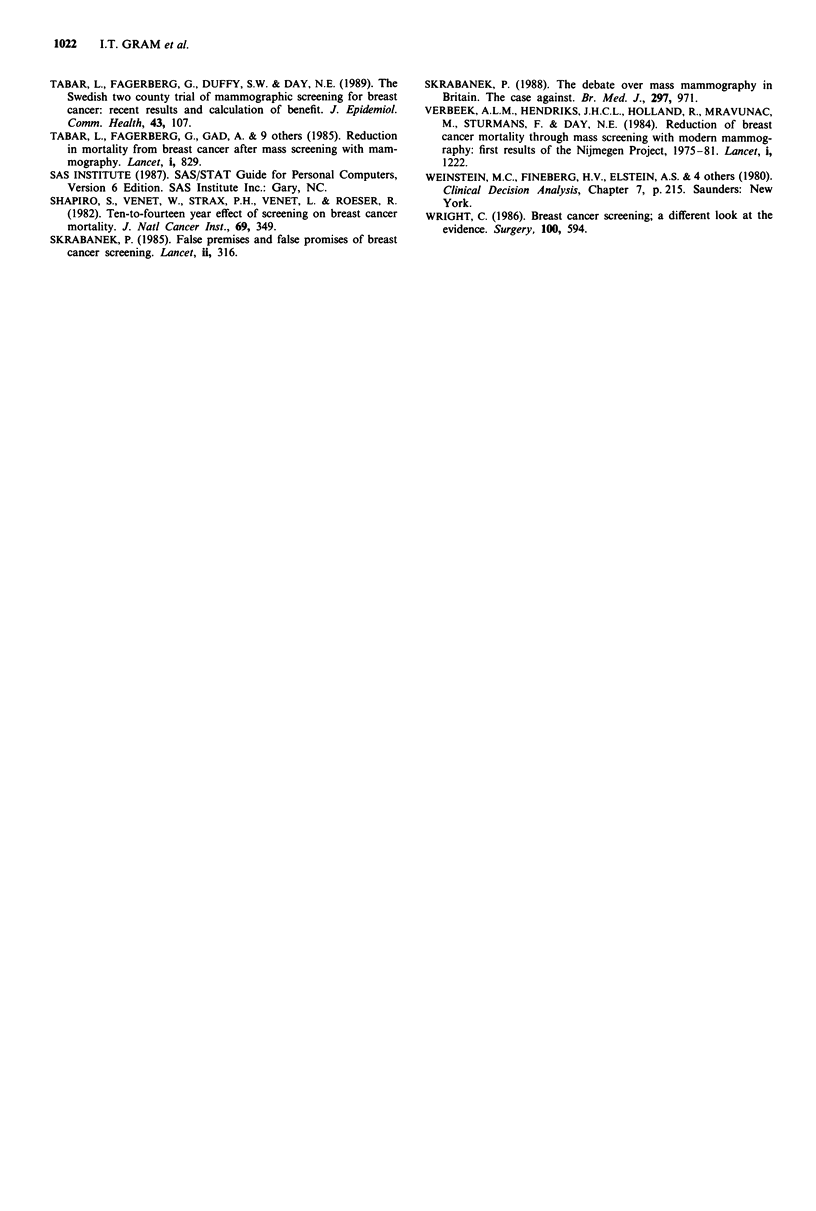


## References

[OCR_00649] Baines C. J., To T., Wall C. (1990). Women's attitudes to screening after participation in the National Breast Screening Study. A questionnaire survey.. Cancer.

[OCR_00658] Collette H. J., Day N. E., Rombach J. J., de Waard F. (1984). Evaluation of screening for breast cancer in a non-randomised study (the DOM project) by means of a case-control study.. Lancet.

[OCR_00664] Dean C., Roberts M. M., French K., Robinson S. (1986). Psychiatric morbidity after screening for breast cancer.. J Epidemiol Community Health.

[OCR_00669] Devitt J. E. (1989). False alarms of breast cancer.. Lancet.

[OCR_00671] Eddy D. M., Hasselblad V., McGivney W., Hendee W. (1988). The value of mammography screening in women under age 50 years.. JAMA.

[OCR_00678] Ellman R., Angeli N., Christians A., Moss S., Chamberlain J., Maguire P. (1989). Psychiatric morbidity associated with screening for breast cancer.. Br J Cancer.

[OCR_00681] Gram I. T., Lund-Larsen P. G., Rosenlund A. F., Størmer J. (1989). Mammografiscreening i Tromsø. Gjennomføring og resultat av den første mammografiscreening i Norge.. Tidsskr Nor Laegeforen.

[OCR_00686] Palli D., Del Turco M. R., Buiatti E., Carli S., Ciatto S., Toscani L., Maltoni G. (1986). A case-control study of the efficacy of a non-randomized breast cancer screening program in Florence (Italy).. Int J Cancer.

[OCR_00709] Shapiro S., Venet W., Strax P., Venet L., Roeser R. (1982). Ten- to fourteen-year effect of screening on breast cancer mortality.. J Natl Cancer Inst.

[OCR_00714] Skrabanek P. (1985). False premises and false promises of breast cancer screening.. Lancet.

[OCR_00718] Skrabanek P. (1988). The debate over mass mammography in Britain. The case against.. BMJ.

[OCR_00694] Tabar L., Fagerberg G., Duffy S. W., Day N. E. (1989). The Swedish two county trial of mammographic screening for breast cancer: recent results and calculation of benefit.. J Epidemiol Community Health.

[OCR_00700] Tabár L., Fagerberg C. J., Gad A., Baldetorp L., Holmberg L. H., Gröntoft O., Ljungquist U., Lundström B., Månson J. C., Eklund G. (1985). Reduction in mortality from breast cancer after mass screening with mammography. Randomised trial from the Breast Cancer Screening Working Group of the Swedish National Board of Health and Welfare.. Lancet.

[OCR_00722] Verbeek A. L., Hendriks J. H., Holland R., Mravunac M., Sturmans F., Day N. E. (1984). Reduction of breast cancer mortality through mass screening with modern mammography. First results of the Nijmegen project, 1975-1981.. Lancet.

[OCR_00734] Wright C. J. (1986). Breast cancer screening: a different look at the evidence.. Surgery.

